# Proof-of-concept study of a small language model chatbot for breast cancer decision support – a transparent, source-controlled, explainable and data-secure approach

**DOI:** 10.1007/s00432-024-05964-3

**Published:** 2024-10-09

**Authors:** Sebastian Griewing, Fabian Lechner, Niklas Gremke, Stefan Lukac, Wolfgang Janni, Markus Wallwiener, Uwe Wagner, Martin Hirsch, Sebastian Kuhn

**Affiliations:** 1 Institute for Digital Medicine, University Hospital Giessen and Marburg, Philipps-University Marburg, Marburg, Germany; 2 Stanford Center for Biomedical Informatics Research, Stanford University School of Medicine, Palo Alto, CA USA; 3 Institute for Artificial Intelligence in Medicine, University Hospital Giessen and Marburg, Philipps-University Marburg, Marburg, Germany; 4 Marburg Gynecological Cancer Center, Giessen and Marburg University Hospital, Philipps-University Marburg, Marburg, Germany; 5 Department of Obstetrics and Gynecology, University Hospital Ulm, University of Ulm, Ulm, Germany; 6 Halle Gynecological Cancer Center, Halle University Hospital, Martin-Luther-University Halle-Wittenberg, Halle (Saale), Germany; 7 Commission Digital Medicine, German Society for Gynecology and Obstetrics (DGGG), Berlin, Germany

**Keywords:** Artificial intelligence, Large language model, Small language model, Breast cancer, Clinical oncology

## Abstract

**Purpose:**

Large language models (LLM) show potential for decision support in breast cancer care. Their use in clinical care is currently prohibited by lack of control over sources used for decision-making, explainability of the decision-making process and health data security issues. Recent development of Small Language Models (SLM) is discussed to address these challenges. This preclinical proof-of-concept study tailors an open-source SLM to the German breast cancer guideline (BC-SLM) to evaluate initial clinical accuracy and technical functionality in a preclinical simulation.

**Methods:**

A multidisciplinary tumor board (MTB) is used as the gold-standard to assess the initial clinical accuracy in terms of concordance of the BC-SLM with MTB and comparing it to two publicly available LLM, ChatGPT3.5 and 4. The study includes 20 fictional patient profiles and recommendations for 5 treatment modalities, resulting in 100 binary treatment recommendations (recommended or not recommended). Statistical evaluation includes concordance with MTB in % including Cohen’s Kappa statistic (κ). Technical functionality is assessed qualitatively in terms of local hosting, adherence to the guideline and information retrieval.

**Results:**

The overall concordance amounts to 86% for BC-SLM (κ = 0.721, *p* < 0.001), 90% for ChatGPT4 (κ = 0.820, *p* < 0.001) and 83% for ChatGPT3.5 (κ = 0.661, *p* < 0.001). Specific concordance for each treatment modality ranges from 65 to 100% for BC-SLM, 85–100% for ChatGPT4, and 55–95% for ChatGPT3.5. The BC-SLM is locally functional, adheres to the standards of the German breast cancer guideline and provides referenced sections for its decision-making.

**Conclusion:**

The tailored BC-SLM shows initial clinical accuracy and technical functionality, with concordance to the MTB that is comparable to publicly-available LLMs like ChatGPT4 and 3.5. This serves as a proof-of-concept for adapting a SLM to an oncological disease and its guideline to address prevailing issues with LLM by ensuring decision transparency, explainability, source control, and data security, which represents a necessary step towards clinical validation and safe use of language models in clinical oncology.

**Supplementary Information:**

The online version contains supplementary material available at 10.1007/s00432-024-05964-3.

## Introduction

Breast cancer (BC) is the most common oncological disease and one of the leading causes of death in women worldwide, with over 2.2 million new cases and 660,000 deaths each year (Ferlay et al. 2024). Extensive research has contributed to improved treatment possibilities that have significantly increased survival rates in recent decades (Taylor et al. 2023). Due to the persistently high burden of disease, a wide range of research efforts continue to expand the spectrum of breast cancer diagnosis and treatment (McIntosh et al. 2023). Recent innovations in precision oncology are catalyzing a paradigm shift in the management of breast cancer and gynecological cancers, facilitated by enhancements in diagnostic and therapeutic modalities (The Lancet Regional Health – Western Pacific 2024). Enhanced genomic profiling capabilities, i.e., next-generation sequencing (NGS) and circulating tumor DNA analyses via liquid biopsies, enable the detailed characterization of oncogenic drivers (Colomer et al. 2023; Baca et al. 2023; Gremke et al. 2024). At the same time, there has been a refinement in the application of targeted therapeutic agents, particularly with the growing availability of antibody-drug conjugates (ADCs) (Dumontet et al. 2023). The utilization of other recombinant humanized monoclonal antibodies has been optimized to target HER2-positive breast cancer phenotypes, enabling the precise ablation of tumor cells overexpressing the HER2 receptor (Pritchard et al. 2006). Additionally, poly (ADP-ribose) polymerase (PARP) inhibitors have demonstrated efficacy in tumors harboring BRCA1/2 mutations, disrupting DNA damage response pathways critical for tumor cell survival (Tutt et al. 2021). In addition, the inclusion of immune checkpoint inhibitors, such as pembrolizumab, has introduced immunomodulatory approaches for the treatment of subtypes such as triple negative breast cancer (TNBC) (Schmid et al. 2020). Current data from the UK 100,000 Genome Project confirm breast cancer to be particularly advantageous to biomarker-directed therapy in comparison to other oncological entities, reporting an overall prevalence of 49% of 1 or more mutations in a target gene (Sosinsky et al. 2024). These developments highlight a significant potential within gynecologic oncology to guide the ongoing development and future refinement of precision oncology approaches (Basu et al. 2018).

This progress in diagnostics and therapy is accompanied by a wealth of multimodal treatment and diagnostic data as well as research findings of increasing complexity that are gradually exceeding the limits of human cognitive processing (Johnson et al. 2021; Porter et al. 2023). A simplified keyword search for “breast cancer” in the PubMed^®^ (US National Institute of Health, Bethesda, MD, USA, performed on May 10th, 2024) identifies 9,269 breast-cancer-related publications in the year of 2003, whereas two decades on, the annual output of corresponding articles has increased by over threefold. Estimates of the doubling time of medical knowledge amounted to 50 years in the 1950s, which has shortened to under three months in this decade (Densen 2011). Nevertheless, the concept of doubling time in medical knowledge, while useful for illustrating rapid advancements, must be interpreted with caution due to its oversimplification and inaccuracies in measuring the complex, varied, and technologically influenced growth of medical information. The first official interdisciplinary guideline issued in 2004 by the German Cancer Society (Deutsche Krebsgesellschaft, DKG) and the German Society for Gynecology and Obstetrics (Deutsche Gesellschaft für Gynäkologie und Geburtshilfe, DGGG) spanned 172 pages and cited 781 references to primary publications (Deutsche Krebsgesellschaft 2004). In contrast, the most recent version from 2021 encompasses 467 pages and references to 1620 primary sources (Leitlinienprogramm Onkologie 2021). Guideline organizations and medical societies are faced with an increasingly difficult challenge of consolidating a flood of scientific evidence into guidelines, keeping them up to date with regular revised versions, and efficiently distributing them to their members and clinical practitioners (Boca et al. 2018). Vice versa, physicians are confronted with ever-longer and more complex guidelines that they must navigate for clinical decision-making, to align treatments with the current state of scientific knowledge (Meskó and Görög 2020; Porter et al. 2023).

Artificial Intelligence (AI), in particular Large Language Models (LLMs), is emerging as a valuable tool to augment human medical intelligence to successfully process large volumes of data and textual information (Johnson et al. 2021; Benary et al. 2023; Maslej et al. 2024). The Artificial Intelligence Index Report of 2024 points out that AI has surpassed human capabilities across a variety of tasks, including image classification, visual reasoning and language understanding (Maslej et al. 2024). In 2023, the United States Food and Drug Administration (FDA) registered a total of 843 authorized AI-related medical devices, marking a 35.5% increase from the previous year (U.S. Food & Drug Administration 2023). This growth reflects a significant rise from just three FDA-approved AI medical devices in 2013 to 221 in 2023 (U.S. Food & Drug Administration 2023). There is growing confidence that leveraging the potential of AI in the medical field can accelerate research and help to close the emerging gap between scientific evidence and clinical practice, leading to more personalized and evidence-based treatment approaches (Basu et al. 2018; Subbiah 2023; The Lancet Regional Health – Western Pacific 2024).

In terms of breast cancer care, exploratory studies on LLMs report promising performance in information extraction from clinical texts, guideline-based question answering and clinical decision support (Benary et al. 2023; Sorin et al. 2024). Nevertheless, the use of LLMs in clinical care is currently restrained by concerns over the control and reliability of the sources used for decision-making, explainability of the decision-making process and health data security (Sorin et al. 2024). Small Language Models (SLMs) including Microsoft’s Phi and Orca, Mistral’s Mixtral-8 × 7B, or Google’s Gemini Nano, are under consideration to address these challenges (Microsoft Co., Redmond, WA, USA; Mistral AI SAS, Paris; France; Google LLC, Mountain View, CA, USA) (Dhunoo 2024). Due to their adaptability and the possibility of local server hosting, SLMs are gaining attention for their application in healthcare (Schick and Schütze 2020; Guo et al. 2023). These models show promise to be tailored to the needs of patient- or disease-specific care pathways by adapting and focusing them on high-quality, evidenced resources (Dhunoo 2024).

Therefore, this study seeks to develop a technological model that adapts an open-source SLM to operate locally and to ensure that it remains explainable in decision-making while being based on evidence from the German breast cancer guidelines. It evaluates the SLM design concept in a preclinical simulation to derive conclusions on initial clinical accuracy and technical functionality by comparing 100 binary treatment recommendations of the BC-SLM with the decisions of a conventional gyne-oncology tumor board (gold standard) and two publicly available LLMs (ChatGPT3.5 and 4.0). At an early stage of development, the proof-of-concept study pursues the objective to provide further insights on whether SLM adaption may help to overcome prevailing issues with LLMs and offer guideline organizations or medical societies an affordable solution to tailor language models to specific medical condition while likewise maintaining decision transparency, source control, explainability and data security.

## Methods

### Technological design concept

Local SLMs are often expected to fall short of the potential of server-hosted LLMs due to the reduced parameter size and training data. This could lead to less precise answers, when it comes to complex queries, as they can be found in breast cancer treatment. To address the challenges, new methodologies have been developed to improve the output of these models, making them more transparent and their outputs more explainable. One of the leading strategies for this endeavor is Retrieval-Augmented Generation (RAG). RAG introduces an upstream search-engine into the SLM framework that is designed to interact with a static information database, in this case clinical guidelines. For this project, the clinical breast cancer guideline was sourced from the “oncology guideline program” (Leitlinienprogramm Onkologie) at the Hasso-Plattner-Institute (HPI, Potsdam, Germany) and DKG. These guidelines, which were made machine-readable for semantic annotation, were detailed in their publication, the German Clinical Guideline Corpus for Oncology (GGPONC) (Borchert et al. 2022). The query engine searches this database to find and retrieve only those segments that semantically align with the query posed to the SLM by the practitioner.

**Fig. 1:**
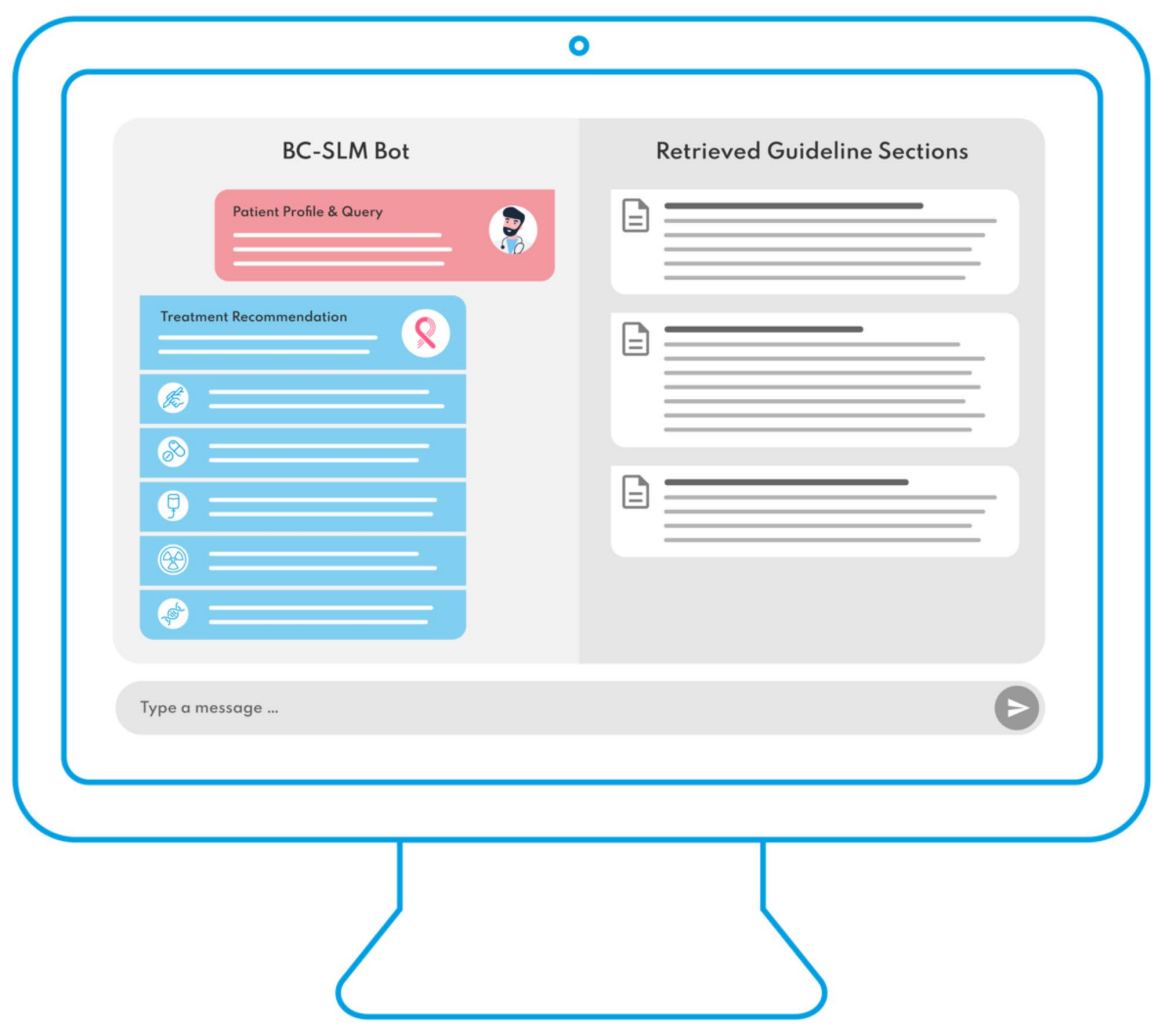
Generic visualization of the BC-SLM frontend

These relevant pieces of information are retrieved from the machine-readable guideline and provided back into the SLM, which is then prompted to generate its response strictly based on this limited data. This mechanism ensures that the SLM’s outputs are not only evidence-based but also explainable in nature. By relying solely on the provided document, the SLM’s responses can be closely controlled, targeting to make the model’s decision-making process more explainable and adherent to the provided source documents. Additionally, the SLM can be liberate enough to summarize the retrieved documents and generate answers still within the context of the user query. This aims to provide the practitioner with a short, yet effective answer to her/his question in combination with traceable referencing to the retrieved sections from the breast cancer guideline, as showcased in Fig. 1, which provides a generic visualization of the chatbot frontend.

In the proposed system, the architecture is designed with a dual retrieval mechanism, in addition to pre-processing and post-processing stages. All instances of SLM involvement are conducted by the Mixtral8 × 7B instruct (Mistral AI SAS, Paris, France) in an unquantized state. This model is capable of running on one A6000 GPU with 48GB of Vram on consumer hardware.

**Fig. 2:**
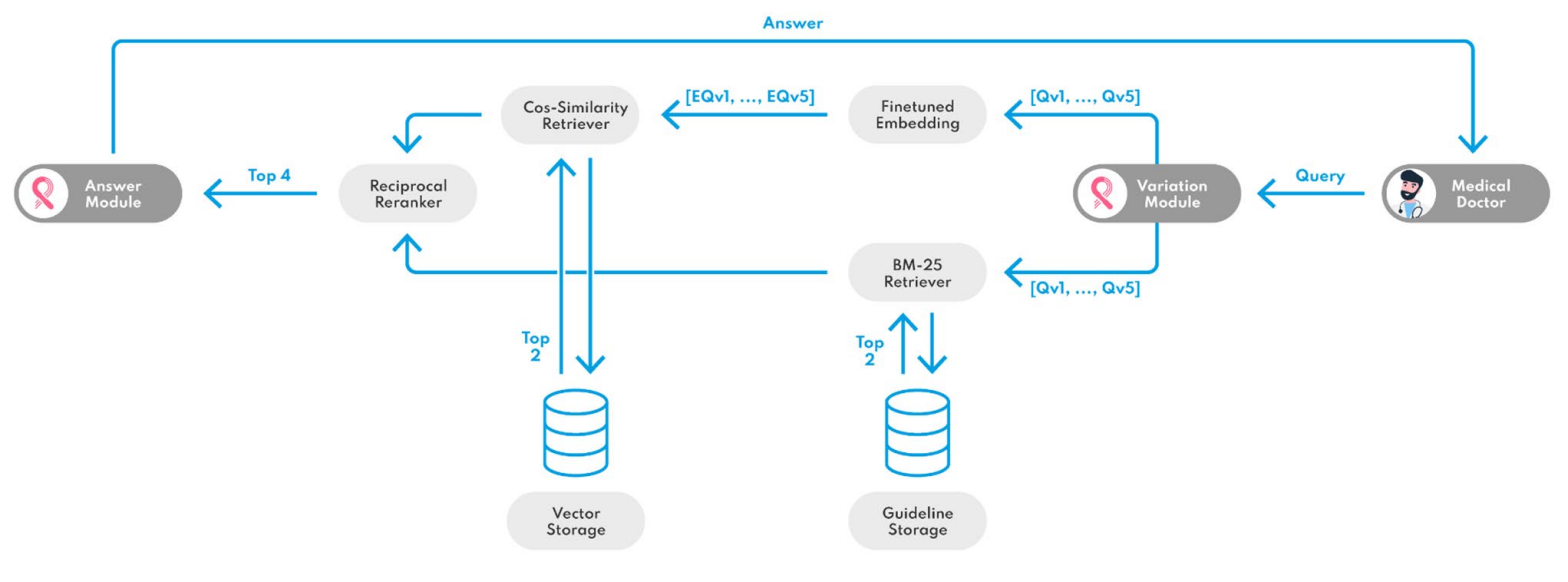
Overview of the technological design concept

Figure 2 provides an overview of the technological design concept. Initially, the user-query undergoes diversification through an interaction with a prompted SLM, enhancing the scope of the semantic search. For the study, five variants of the query, including the original, are being formulated by the SLM. These queries are then processed by two distinct retrieval methods. The first retrieval strategy employs AI-embedding techniques coupled with cosine similarity measures to identify data with closely related semantic content. The second retrieval method employs the BM25 algorithm, a refined form of keyword search that operates independently of embedding techniques. Subsequently, a mutual reranking module systematically reorders the retrieved documents based on their relevance to the initial query. The two most relevant documents are then passed onto a pre-prompted SLM. This model synthesizes the original query with the selected documents using a set of given hyperparameters to constrain its ability to hallucinate and construct a precise and contextually relevant response to the query.

### Preclinical simulation and comparative analysis

**Fig. 3:**
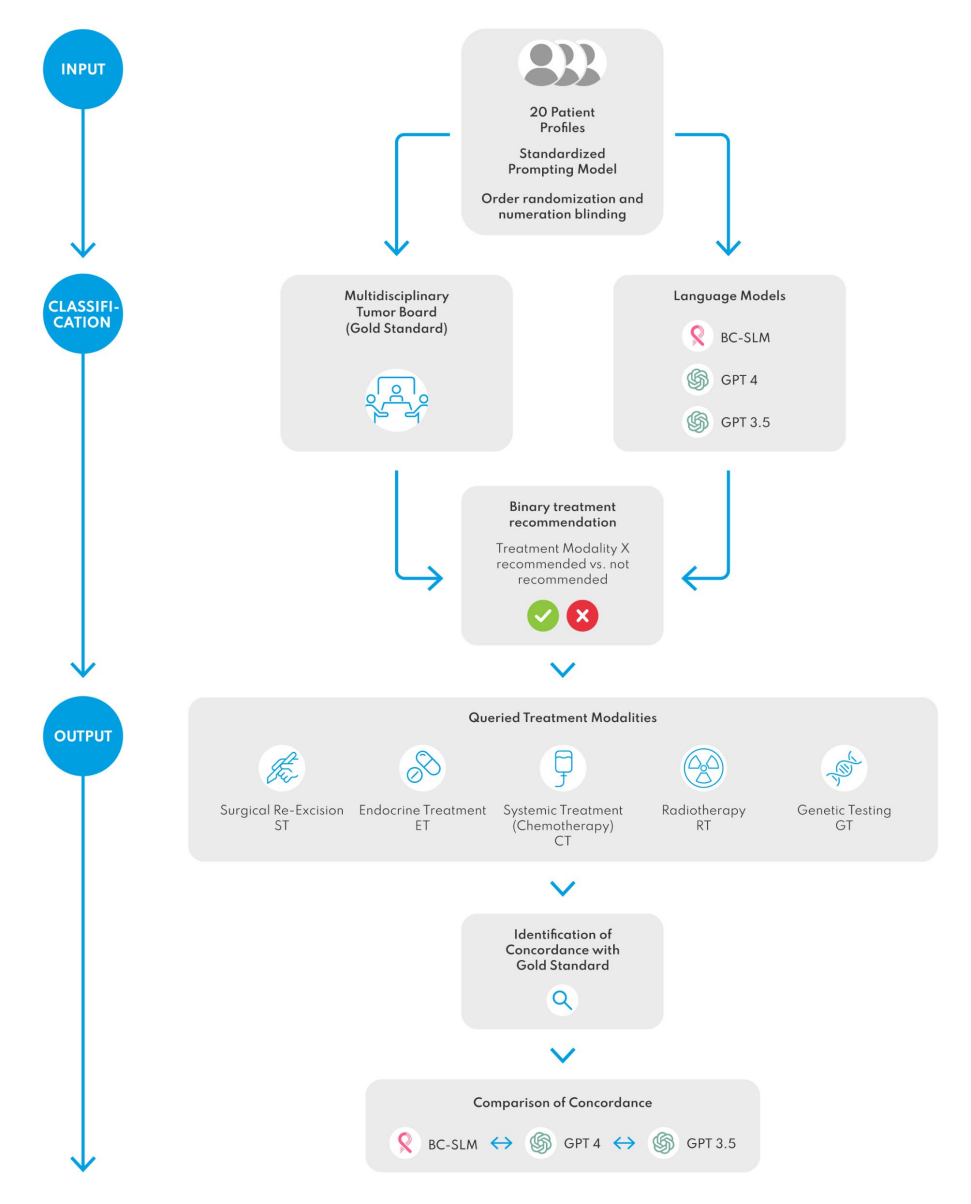
Structure of the preclinical simulation

This proof-of-concept study evaluates the initial clinical accuracy and technical functionality of the developed technological design in a preclinical simulation environment. Therefore, it assesses the concordance of treatment recommendations from the BC-SLM with those made by a conventional gynecological oncology tumor board (considered the gold standard) and compares the results with two publicly available LLMs, ChatGPT3.5 and 4.0 (OpenAI Inc., San Francisco, CA, USA). Testing publicly accessible Large Language Models (LLMs) restricts the study to use fictional patient profiles in order to avoid breaching personal data integrity and not complying with the European General Data Protection Regulation (GDPR) or the German Federal Data Protection Act (DSVGO). Consequently, the study did not use any real patient history, and therefore, an ethics vote was waived by the Research Ethics Committee of Philipps-University Marburg (23–300 ANZ). The study design follows the recommendations by Sorin et al. from a recent literature review on the utilization of LLMs in breast cancer management (Sorin et al. 2024). As such it utilizes 20 breast cancer patient profiles (PP) that comprehensively represent the full spectrum of breast carcinoma subtypes, following the German Association of Gynecology and Obstetrics (DGGG) guideline (version 4.4, May 2021, AWMF-registration number 032/0456OL). This fictional cohort covers diverse immuno- and histopathological subtypes, pre- and postmenopausal status, and includes cases of precancerous and primary metastatic disease (see supplementary material 1 for generic patient profiles). These PP have previously been used and published for evaluation of LLM functionality in the field of breast cancer care (see supplementary material 1). The prompt for treatment recommendations followed a standardized and previously published prompting model, which was assessed as the current best practice by Sorin et al. based on QUADAS-2 assessment (Quality Assessment of Diagnostic Accuracy Studies-2, QUADAS-2), which aims to minimize interpretation bias (see supplementary material 1). Recommendations were requested for the five treatment modalities (TM) of surgical re-excision (ST), endocrine treatment (ET), chemotherapy (CT) as well as radiotherapy (RT). Furthermore, the recommendation for genetic counseling and testing (GT) was inquired. The order of the profiles was randomized and presented in a blinded version without numeration, to ensure that the structured query of the different cancer subtypes did not remain recognizable. The gold standard was established by querying the multidisciplinary gynecological tumor board (MTB) of the certified gynecological cancer center of Marburg University Hospital for treatment recommendations. Afterwards, the BC-SLM and LLMs were equivalently prompted for therapy suggestions. The treatment recommendations were identified in binary manner ({TM} recommended versus {TM} not recommended). Thus, the study involves a total of 100 binary decisions based on the 20 PP across the five TM (20 × 5 = 100). The concordance percentage with the gold standard (MTB) was calculated for each language model, both for overall treatment recommendations (*n* = 100) and for each treatment modality separately (*n* = 20 for each of the 5 modalities: ST, ET, CT, RT, and GT), Cohen´s kappa statistic was included to account for the possibility of agreement occurring by chance and to provide a more reliable measure of concordance. Statistical significance of the Kappa values was assessed with *p*-values, where *p* < 0.001 indicates strong, *p* < 0.01 moderate and *p* < 0.05 low significance. SPSS (Version 29.0.2.0, IBM Corporation, Armonk NY, USA) was used for statistical analysis. Further qualitative assessment involved the confirmation whether the BC-SLM can be hosted on the local computer, as benchmark of localization for data security, can be restricted to the guideline, as a benchmark of source control for evidence-based decision-making, and can be induced to provide the retrieved sections of the guideline, as a benchmark of transparency and explainability of the decision-making process. The structured process of the preclinical simulation is visualized in Fig. 3.

## Results

### Results for overall treatment recommendations and qualitative assessment of the BC-SLM

The study evaluated 100 binary treatment recommendations, assessing their concordance with the gold standard set by the multidisciplinary tumor board (MTB). Concordance in % was highest for ChatGPT-4 at 90.0%, followed by BC-SLM at 86.0%, and ChatGPT-3.5 at 83.0%. Cohen’s Kappa values indicated substantial and significant agreement for all models. The qualitative assessment during the prompting showcases the BC-SLM´s functionality on the local computer while restricting the input resource to the machine-readable German standard of the breast cancer guideline. The SLM provides referencing to the retrieved sections of the guideline used for its decision-making process.

**Table 1:** Results for overall treatment recommendations

	Results for Overall Treatment Recommendations (*n* = 100)		
	BC-SLM	GPT4	GPT3.5
MTB	86.0% (0.721***)	90.0% (0.820***)	83.0% (0.661***)

Explanation of values:

Concordance with multidisciplinary tumor board in % (Cohen´s kappa κ;

with *indicating *p*-value < 0.05, ** indicating *p*-value < 0.01 and *** indicating *p*-value < 0.001)

Abbreviations:

MTB = multidisciplinary tumor board (gold standard), BC-SLM = breast cancer small language model, ST = surgical re-excision, ET = endorince treatment, CT = systemic or chemotherapy, RT = radiotherapy, GT = necessity for genetic counseling

### Results for each treatment modality

All models have high concordance with the MTB across various treatment modalities. For ET, ChatGPT4 showed an 85.0% concordance with a Cohen’s kappa of 0.706, suggesting a significant agreement (*p* < 0.001). In CT, BC-SLM achieved 100% concordance, while ChatGPT4 and 3.5 also demonstrated high concordance, with statistically significant kappa values. A similar pattern is observed RT, where ChatGPT4 reached 100% concordance. For GT, ChatGPT4 achieved an 85.0% concordance and the highest kappa value (0.794, *p* < 0.001), whereas BC-SLM and ChatGPT3.5 had lower concordance rates of 65.0% and 55.0%, with low, non-significant kappa values.

**Table 2:** Results for each treatment modality

	Results for Each Treatment Modality (*n* = 20)				
	ST	ET	CT	RT	GT
BC-SLM	100.0%	80.0% (0.615**)	100.0%	85.0% (0.625**)	65.0% (0.255)
GPT4	95.0% (0.773***)	85.0% (0.706***)	85.0% (0.667**)	100.0%	85.0% (0.794***)
GPT3.5	95.0% (0.773***)	80.0% (0.615**)	90.00% (0.792***)	95.0% (0.857***)	55.0% (0.022)

Explanation of values:

Concordance with multidisciplinary tumor board in % (Cohen´s kappa κ;

with *indicating *p*-value < 0.05, ** indicating *p*-value < 0.01 and *** indicating *p*-value < 0.001)

Abbreviations:

BC-SLM = breast cancer small language model, ST = surgical re-excision, ET = endorince treatment, CT = systemic or chemotherapy, RT = radiotherapy, GT = necessity for genetic counseling

For further details a table with the language models´ concordance with MTB for each treatment modality per patient profile can be found in the supplementary material (see supplementary material 2).

## Discussion

### Main findings

Over the last two decades, significant improvements in diagnostic and therapeutic methods have enhanced treatment outcomes and survival rates in clinical oncology, culminating in a growing body of meaningful scientific evidence (Taylor et al. 2023; Subbiah 2023). Partially automated data processing and preparation with the help of AI is seen as a decisive accelerator in breaking up this flood of scientific knowledge for clinical practitioners (Meskó and Görög 2020; Johnson et al. 2021). Due to their strength in textual processing, Large Language Models (LLMs) are discussed as a meaningful technological solution in addressing this issue (Benary et al. 2023; Sorin et al. 2024). Current barriers to the deployment of LLMs in clinical settings include inadequate control over the sources used for decision-making, missing transparency of the decision-making process and concerns about the security of health data being processed via decentralized international servers (Sorin et al. 2024). Due to their adaptability and the possibility of local server hosting, Small Language Models (SLMs) are gaining attention for their potential in addressing these issues (Schick and Schütze 2020; Dhunoo 2024).

This study is, to our knowledge, the first to adapt an open-source SLM to a clinical oncology guideline. It proves the concept of aligning a SLM with the national evidence consensus for an oncological entity, achieving reliable initial clinical accuracy for breast cancer by providing binary treatment decisions that are consistent with a conventional tumor board’s expert recommendations. Additionally, it achieves concordance levels comparable to publicly available LLMs such as ChatGPT4 and GPT3.5. This finding underscores the technical functionality of the SLM design concept and suggests that SLMs could offer a secure solution for health data processing by operating on local servers or computers. This helps to overcome the previously stated barriers to the deployment of LLMs in clinical care. In line with the Explainable AI (XAI) approach, the decision-making process’s traceability can be enhanced by restricting the model’s decision pathways and narrowing the AI system’s scope (Kundu 2021). Consequently, the developed BC-SLM remains transparent in its decision-making by disclosing the breast cancer guideline sources it references and how these inform its treatment recommendations. This adaptation of an open-source SLM offers a transparent, source-controlled, explainable, and data-secure approach for using language models in clinical oncology, enabling the processing of patient-specific health data in alignment with established national and international diagnostic and treatment standards.

### Further findings

#### Expanding the potential of LLMs to SLMs in breast cancer care

In the previous course of exploration of the practical use of LLMs in breast cancer care, Rao et al. showcased the successful employment of GPT3.5 for radiology imaging evaluations, confirming its value in breast cancer care with regard to mammography analysis (Rao et al. 2023). Haver et al. showcased the capability of a chatbot to educate patients on breast cancer prevention and screenings measures (Haver et al. 2023). Additionally, Choi et al. exhibited the potential of custom prompts for LLMs in retrieving clinical insights from extensive breast cancer patient records, encompassing multimodal data from pathology and ultrasound reports (Choi et al. 2023). In the context of decision-support, Lukac et al. and Sorin et al. have conducted explorative studies to compare the quality of decision-making between GPT3.5 and tumor boards (Lukac et al. 2023; Sorin et al. 2023). Sorin et al.‘s recent review article synthesizes the current literature on the utilization of LLMs in breast cancer management (Sorin et al. 2024). The overview identifies the most promising application areas in breast cancer care in the processing of textual data and disease-related question-answering. However, they conclude that the current level of evidence regarding the deployment of LLMs in breast cancer management remains in an early-stage phase of feasibility exploration, highlighting a critical need for future rigorous clinical validation and continuous monitoring going forward (Sorin et al. 2024). This study ties into these findings by underscoring the potential of language models for textual processing and decision support and expanding these findings from Large to Small Language Models in the field of breast cancer care.

Nevertheless, it is crucial to note in the context of interpretation that this proof-of-concept still represents an early step in the further development of language models in the medical domain. As stated, the findings of this study demonstrate that the adaptation of a SLM may help to overcome prevailing issues with use of LLMs. Nonetheless, it is a proof-of-concept study, which entails significant limitations in the clinical interpretability of the results. An exploratory proof-of-concept study for a health technology tool aims to assess the initial viability, functionality, and potential impact of the tool in a controlled, oftentimes preclinical, setting, providing preliminary insights into its functionality. In contrast, an early feasibility study focuses on evaluating the tool’s safety, usability, and basic efficacy in a real-world context, while a clinical validation study rigorously tests its effectiveness and reliability in a larger, more diverse patient population to establish its clinical value. In the following, we address these limitations of the current state of knowledge and outline how corresponding studies can gradually increase the evidence level in the use of language models in breast cancer care and clinical oncology.

#### Limitations: iterative technological modification towards clinical validation

This study serves as a preclinical proof-of-concept, evaluating a newly developed technological model within a preclinical simulation environment, focusing on its initial clinical accuracy and technical functionality. It is crucial to emphasize that this study does not offer clinical validation for either the performance of LLMs or SLMs. While the results may indicate potential patterns of concordance across different cancer subtypes or stages (e.g., DCIS, TNBC, etc.) and varying levels of agreement for specific treatment modalities (e.g., the relatively low concordance and non-significant Cohen’s kappa for GT), these observations merely suggest possible differences. However, due to the exploratory nature of this proof-of-concept, such patterns are beyond the scope of this evaluation and warrant investigation in future studies. It is known that language models continue to have crucial problems with reliability and reproducibility (Sorin et al. 2024). Thus, it should not be directly inferred from the results that one model is better than the other or performs better or worse for different treatment modalities and different tumor stages or subtypes. As described by Sorin et al. in the recent literature review, the exploration of language models in breast cancer care is in an early stage of development but requires ongoing supervision and monitoring as the practical application of language models in clinical oncology is evolving (Sorin et al. 2024). Following technological refinement, future feasibility and clinical validation studies should include study designs that incorporate larger-scale study populations and more diverse settings to allow for comprehensive validation. Additionally, preclinical studies should include simulation settings with various users assessing user-specific aspects and hybrid decision-making. Important limitations are explained in more detail below in order to avoid misinterpreting the results by drawing conclusions that are outside the scope of this study.

Firstly, the study uses a small number of patient profiles for testing. This process was chosen to comprehensively cover the spectrum of patho- and immunomorphological types of breast carcinoma in accordance to Sorin et al.´s recommendations and findings of previous studies (Sorin et al. 2024). Nevertheless, neither does this approach allow for a conclusive comparison of treatment modalities or cancer subtypes (i.e., DCIS versus invasive, Her2 positive versus Her2 negative, Luminal A versus Luminal B, TNBC versus HR + or early-stage versus metastatic carcinoma) nor one should expect the cases to produce, for instance, an age distribution that aligns with the epidemiological or demographic data of a specific population, e.g. on national level for a specific country. This consideration is crucial for the subsequent feasibility studies and the comprehensive clinical validation of the technology. A crucial step in further developing the system will be to test it with a more diverse or nationally representative study cohort, encompassing hundreds to thousands of patient profiles. This will provide more robust evidence on identifying particularly useful applications of language models in clinical oncology, specifically determining whether language models offer significant performance benefits for certain treatment modalities or specific cancer subtypes and stages. Secondly, the study establishes the recommendations of a singular multidisciplinary tumor board as the gold standard. Several international research groups, i.e. the EURECCA and EUSOMA networks, have carried out extensive observational studies, uncovering significant differences in the treatment choices and outcomes for breast cancer between certified centers (Derks et al. 2018; van Walle et al. 2023). There is a significant scope for decision-making in breast cancer treatment and, therefore, future studies should incorporate a larger group of national and international centers to enable a more balanced basis for comparison (Derks et al. 2018). Thirdly, the study is based on the German breast cancer guideline and was carried out in a German gynecological center. Nevertheless, there is significant variability in national standards and guidelines for breast cancer care decision-making. The results should therefore be interpreted on the basis of German standards, although the intuitive interpretation may vary depending on the international background of the reader.

#### Research perspective: feasibility of guideline navigation and the perspective on SLM-powered oncological decision support

Facing the growing body of meaningful evidence in breast cancer care, clinical practitioners are confronted with increasingly lengthy and complex guidelines that they can use to guide their clinical decision-making in order to bring treatments in line with the current state of scientific knowledge (Porter et al. 2023). To improve accessibility, guideline organizations and medical societies are investing considerable financial and personnel resources in synthesizing this extensive research into guidelines (Boca et al. 2018). Regarding German gynecological oncology, this is traced back to extensive evidence syntheses, i.e., for breast (467 pages) or endometrial cancer (354 pages) (Leitlinienprogramm Onkologie 2021; Leitlinienprogramm Onkologie 2023). Beyond that, further oncological specialties offer even more complex evidence synthesis, e.g., for lung (592 pages) and prostate cancer (473 pages) (Leitlinienprogramm Onkologie 2024a, b). These guidelines, which incorporate references to up to thousands of primary publications in their metadata, e.g., over 1600 primary publications for the lung cancer guideline, need to be updated on a regular basis to reflect the rapid advancement of medical knowledge.

The application of SLMs may offer a prospective solution to bridge the gap between cutting-edge oncological evidence and clinical practice. The study showcases how the localized, guideline-based chatbot provides an interactive platform that exceeds a simplified keyword research and that responds to specific queries, thereby facilitating quick navigation to pertinent sections within the extensive 467-page German breast cancer guideline. The future adaptation of guideline based SLMs may provide an affordable and feasible solution that can help lower the information asymmetry between state-of-the-art oncological research and clinical oncology by efficient guideline navigation. In qualitative assessment, the BC-SLM strictly conforms to the breast cancer treatment recommendations of the DGGG guideline while all data processing occurs on the local computer in the hospital. This can enable a transparent and explainable decision-making process in alignment with the AIX approach. Users can understand the decision-making process by consulting the specified guideline sections or by engaging with the chatbot. A necessity to build trust between the medical user and the AI (Kundu 2021). Based on the simplified architecture of the SLM, the clinical outputs become more transparent and interpretable. The possibility to focus the SLM on preselected evidence and high-quality scientific data allows for the adaption of the model to a personalized and disease-specific patient pathway. A future area of exploration might be the dynamic coupling of the BC-SLM to existing machine-readable guideline corpora. For example, by deploying an application programming interface to national or international guideline apps, e.g., “Oncology Guidelines App” of the oncology guideline program (Leitlinienprogramm Onkologie) of the German Cancer Society (Deutsche Krebsgesellschaft, DKG) (Borchert et al. 2022), this could allow for access to the most current evidence synthesis and the underlying metadata with its primary literature. In perspective, this may also provide a valuable foundation to steer device modification to explore more reliable oncological decision support. Based on the findings of the study, a future area of exploration might be the integration of predefined treatment algorithms, knowledge graphs and doctoral decision trees of the breast cancer patient pathway into the newly developed SLM design concept to minimize the prevailing challenge of language model hallucination and optimize decision reliability and accuracy (Ji et al. 2022; Benary et al. 2023; Sorin et al. 2024). Another area of exploration for SLM-powered decision support is its integration into preexisting care processes and information technology infrastructures. To enhance patient-centricity, dynamic coupling of a tailored SLM with digital health or telemonitoring applications could enable the incorporation of more personalized, multimodal real-world data (e.g., continuous vital parameters, patient-reported outcomes on psychosocial factors, environmental data) into its decision-making process. Additionally, SLMs could be integrated with existing data infrastructures within hospital information systems, such as electronic health records, histopathology, laboratory results and imaging data. Enabling future models to integrate a patient’s comprehensive history by multimodal data integration would allow these models to consider more patient-specific criteria, thereby providing more personalized decision support. At the same time, this integration would provide more efficient support to clinicians by reducing the need for manual data entry and automating data processing within clinical data infrastructures.

## Conclusion

This study provides a preclinical proof-of-concept for the adaption of an open-source SLM in the field of clinical oncology. It demonstrates initial clinical accuracy by providing binary treatment decisions for breast cancer that align with the expert recommendations of a conventional tumor board. Furthermore, it achieves concordance levels comparable to other publicly available LLMs, such as ChatGPT4 and ChatGPT3.5. The adaption of a SLM to address the specific characteristics of a medical condition, as shown by focusing the SLM Mixtral-8 × 7B on the machine-readable German breast cancer guideline, is functional. The technological design concept allows for localization of the language model while likewise restricting it to a preselected evidence synthesis and leveraging explainability of the AI decision-making process. At an early stage of development, the study demonstrates that SLM adaption may offer guideline organizations and medical societies an affordable solution to tailor language models to a specific medical condition or patient pathway while likewise ensuring decision transparency, explainability, source control and data security. This is a necessary step in the technological refinement of language models and may guide further modifications towards their clinical validation and subsequent safe use in clinical oncology.

## Electronic supplementary material

Below is the link to the electronic supplementary material.

Supplementary Material 1

Supplementary Material 2

## Data Availability

Data is provided within the manuscript or supplementary information files. Further datasets generated during and analyzed during the current study are available from the corresponding author on reasonable request.
