# Supplementary material for: Proof-of-concept study of a small language model chatbot for breast cancer decision support – a transparent, source-controlled, explainable and data-secure approach
Source: J Cancer Res Clin Oncol. 2024 Oct 9;150(10):451. doi: 10.1007/s00432-024-05964-3 (PMC11464535; doi:10.1007/s00432-024-05964-3)

Supplementary Material 1

Generic Patient Profiles, PP1-20

Patient Profiles				
Immunohistochemical and molecular subtypes	Postmenopausal		Premenopausal	
	Nodal negative	Nodal positive	Nodal negative	Nodal positive
Luminal A	P1	P2	P3	P4
Luminal B	P5	P6	P7	P8
Her2 positive	P9	P10	P11	P12
Triple negative	P13	P14	P15	P16
DCIS	P17		P18	
DCIS with narrow resection margin	P19			
Inflammatory breast cancer				P20

Patient Profile 1-10

P1-10	P1	P2	P3	P4	P5	P6	P7	P8	P9	P10
Patient Profiles	Postmenopausal Luminal A N-	Postmenopausal Luminal A N+	Premenopausal Luminal A N-	Premenopausal Luminal A N+	Postmenopausal Luminal B Her2- N-	Postmenopausal Luminal B Her2- N+	Premenopausal Luminal B Her2- N-	Premenopausal Luminal B Her2+ N+	Postmenopausal Her2+ ER/PR- N-	Postmenopausal Her2+ ER/PR- N+
Age	62	61	50	45	62	58	40	35	58	65
Menopause Status	post	post	pre	pre	post	post	pre	pre	post	post
ECOG	0	0	1	1	0	1	0	0	1	2
Previous Illness	Bronchial asthma (no long-term therapy, acute therapy with inhaled corticosteroids and formoterol), arterial hypertension (with antihypertensive triple combination of diuretic, calcium antagonist and AT II antagonist)	Hypothyroidism (with L-thyroxine medication)	Relapsing remitting multiple sclerosis (last episode 5 years ago, no long-term medication)	HELLP Syndrome at first pregnancy at age of 34	Diabetes mellitus type 1, arterial hypertension (with ACE inhibitor medication), hemorrhoids	Crohn's disease (with continuous therapy with TNF-alpha inhibitors)	Deep vein thrombosis at age 25 while on contraceptive medication, heterozygous factor V Leiden	Colitis ulcerosa, Hashimoto-thyroiditis (with L-thyroxine medication)	COPD GOLD B (with inhaled long-acting muscarinic receptor antagonists and inhaled long-acting ß2 sympathomimetics medication)	Atrial fibrillation (with direct oral anticoagulant and beta-blocker medication), pulmonary artery embolism at the age of 65 following immobilization during right-sided total hip arthroplasty
Previous surgical treatment	Transverse laparotomy for hysterectomy because of hypermenorrhea and uterine myomatosus at age of 42, laparoscopic cholecystectomy at the age of 45, open appendectomy at the age of 29	Open cholecystectomy at the age of 35, breast-conserving tumorectomy for right-sided fibroadenoma at the age of 32, uterine curettage after early abortion at age of 20	Tonsillectomy in childhood, open appendectomy for complicated appendicitis without free perforation at the age of 27	Postpartum cardiomyopathy with intensive care ECMO support, Roux-Y gastric bypass for obesity (BMI 50) at the age of 32	Mamma abscess cleavage on the right side at the age of 35, open hemorrhoidectomy according to Milligan-Morgan at the age of 40	Bowel-sparing resection for ileum stenosis at the age of 35, open appendectomy at the age of 25, longitudinal laparotomy for mechanical ileus at the age of 55	Open appendectomy at the age of 28	Laparoscopy for cyst extirpation of left ovarian cyst at age 30	none	Right-sided total hip arthroplasty at the age of 65
Birth history	1 vaginal birth at age of 32, 1 cesarean at the age of 34, 1 early abortion at the age of 30	4 vaginal births at the age of 25, 27, 29 and 30, 1 early abortion at the age of 20	no prior birth	2 cesareans at the age of 34 and 38	4 vaginal births at the age of 18, 20, 28 and 30	no prior birth	1 vaginal birth at the age of 39	no prior birth	2 vaginal births at the age of 28 and 30	2 vaginal births at the age of 23 and 30 and 1 cesarean at the age of 35
Oncological family history	Maternal aunt with colon cancer at the age of 62	Maternal female cousin with hodgkin lymphoma at the age of 30	no prior oncological family history	Paternal uncle with prostate cancer at the age of 65	Paternal uncle with colon-cancer at the age of 40, paternal grandfather with colon-cancer at the age of 60, paternal cousin with colon cancer at the age of 35	Maternal grandmother with breast cancer at the age of 80	Sister-in-law with breast cancer at the age of 30	Paternal grandmother with breast cancer at the age of 70, paternal aunt with breast cancer at the age of 50, maternal uncle with pancreatic cancer at the age of 60	Maternal grandmother with endometrial cancer at the age of 75, mother with bile duct carcinoma at the age of 60	Sister with childhood acute lymphoblastic leukemia, father with gastric carcinoma at the age of 50
Previous surgical treatment	BCT+SLN right	BCT+SLN left	BCT+SLN right	BCT+SLN left	BCT+SLN right	BCT+SLN left	BCT+SLN left	BCT+SLN right	BCT+SLN left	MT+SLN right
TNM	pT1bN0MX	pT2(2)pN1aM0	pT1apN0MX	pT1cpN1aM0	pT3pN0M0	pT3(3)pN1aM0	pT2pN0M0	pT2pN1cM0	pT1apN0M0	pT3pN1aM0
Resection margin	R0, 5mm	R0, 6mm	R0, 1mm	R1 on lateral aspect	R0, 0.1mm	R0, 7mm	R1 on lateral aspect	R0, 2mm	R0, 0.05mm	R0, 10mm
Histological subtype	NST	Invasive-lobular	Mucinous	NST	Invasive-lobular	NST	Tubular	Invasive-lobular	NST	NST
Grading	G1	G2	G1	G2	G1	G2	G2	G3	G2	G2
UL/BL	Unilateral	Unilateral	Unilateral	Unilateral	Unilateral	Unilateral	Unilateral	Unilateral	Unilateral	Unilateral
MF/MC	Monofocal and -centric	Monocentric and multifocal, 2 foci	Monofocal and -centric	Monofocal and -centric	Monofocal and -centric	Monocentric and multifocal, 3 foci	Monofocal and -centric	Monofocal and -centric	Monofocal and -centric	Monofocal and -centric
ER	95%	85%	95%	100%	80%	75%	90%	75%	5%	0%
PR	80%	80%	90%	100%	75%	90%	50%	75%	1%	0%
Her2	Negative (IHC 0)	Negative (IHC 1+)	Negative (IHC 0)	Negative (IHC 0)	Negative (IHC 1+)	Negative (IHC 0)	Negative (IHC 2+, ISH negative)	Positive (IHC 3+)	Positive (ISH positive)	Positive (ICH 3+)
Ki-67	10%	15%	8%	10%	35%	28%	30%	40%	20%	35%

N+/-= nodal positive or negative, Her2+/-= Her2 positive or negative, BCT= breast-conserving tumorectomy, SLN= sentinellymphnodectomy, MT= mastectomy, UL/BL= uni- versus bilaterality, MF/MC= multifocality or -centricity, ER= estrogen receptor, PR= progesterone receptor, Her2= Her2 status, Ki-67= Ki-67-proliferation-index

Patient Profile 11-20

P11-20	P11	P12	P13	P14	P15	P16	P17	P18	P19	P20
Patient Profiles	Premenopausal Her2+ ER/PR- N-	Premenopausal Her2+ ER/PR- N+	Postmenopausal Triple Negative N-	Postmenopausal Triple Negative N+	Premenopausal Triple Negative N-	Premenopausal Triple Negative N+	Postmenopausal DCIS, clear resection margin	Premenopausal DCIS, clear resection margin	Postmenopausal DCIS, narrow resection margin	Inflammatory Breast Cancer
Age	32	42	56	65	29	35	70	38	72	36
Menopause status	pre	pre	post	post	pre	pre	post	pre	post	pre
ECOG	0	0	1	1	0	0	2	0	1	0
Previous Illness	Insulin-dependent gestational diabetes during the first pregnancy at the age of 20, postpartum depression at the age of 20	Pulmonary artery embolism after pelvic vein thrombosis at the age of 26, antiphospholipid syndrome with anti-cardiolipin antibodies (with permanent oral anticoagulation with phenprocoumon)	Insulin-dependent diabetes mellitus type 2, obesity with BMI of 43, obstructive sleep apnea syndrome, secondary arterial hypertension (with ACE inhibitor medication)	Addison's disease (currently under hydro- and fludrocortisone medication)	none	Paranoid schizophrenia (under stable condition with current olanzapine medication)	Arterial hypertension (with with AT II antagonist medication)	AV-node re-entry tachycardia (with beta-blocker medication)	Chronic lymphocytic leukemia Stadium A	none
Previous surgical treatment	none	none	Abdominoplasty at the age of 50, bilateral mammary reduction mammoplasty for mammary hypertrophy at the age of 35	Total knee replacement on the left side at the age of 50	none	none	Vaginal hysterectomy with bilateral adnexectomy for uterine prolapse at the age of 55, transcatheter aortic valve implantation due to aortic valve stenosis at the age of 69	none	Total shoulder arthroplasty on the left side	none
Birth history	1 vaginal birth at the age of 20	6 early abortions between the age of 20 and 26	3 vaginal births at the age of 20, 21 and 25	3 cesareans at the age of 25, 28 and 35	no prior birth	no prior birth	2 vaginal births at the age of 16 and 20	1 cesarean at the age of 36	1 vaginal birth at the age of 22	no prior birth
Oncological family history	Father with bronchial carcinoma at the age of 60, sister with osteosarcoma at the age of 18	Father with colon cancer at the age of 45, paternal grandmother with endometrial cancer at the age of 65, paternal uncle with urothelial carcinoma of the renal pelvis at the age of 55	Mother with breast cancer at the age of 40	Paternal grandmother with pancreatic cancer at the age of 59, maternal aunt with colon cancer at the age of 60	Mother with breast cancer at the age of 65, maternal grandmother with breast cancer at the age of 70	Maternal grandmother with endometrial cancer at age of 60, paternal uncle with rectum carcinoma at the age of 50	1 sister with peritoneal cancer at the age of 60, maternal grandmother with ovarian cancer at the age of 65	Paternal grandfather with prostate cancer at the age of 65, mother with chronic myeloid leukemia at the age of 70	Father with colon cancer at age 55	no cancer history
Previous surgical treatment	BCT+SLN left	BCT+SLN right	BCT+SLN left	none so far	none so far	none so far	MT right	BCT left and right	BCT+SLN left	none so far
TNM	pT2pN0M0	pT2pN1M0	pT1apN0M0	cT3pN+pM1 (OSS)	cT2cN0M0 on left side and cT1bcN0M0 on right side	cT2pN+pM1 (HEP)	pTis (size of the lesion 4.3 cm)	pTis on left and right side (size of the lesions: 2.3 cm on left and 3.2 cm on right side)	pTis (size of lesion 1.5 cm)	cT4dpN+M0
Resection margin	R0, 4mm	R0, 2mm	R0, 1mm	not applicable	not applicable	not applicable	R0, 10mm	R0, 4 mm on left, 5 mm on right side	R0, 0.01mm	not applicable
Histological subtype	NST	NST	NST	NST	NST on left and right side	NST	not applicable	not applicable	not applicable	NST, inflammatory breast cancer with lymphangiosis carcinomatosa
Grading	G2	G3	G2	G3	G3	G3	not applicable	not applicable	not applicable	G3
UL/BL	Unilateral	Unilateral	Unilateral	Unilateral	Bilateral	Unilateral	Unilateral	Bilateral	Unilateral	Unilateral
MF/MC	Monofocal and -centric	Monofocal and -centric	Monofocal and -centric	not applicable	not applicable	not applicable	Monofocal and multicentric, 2 centers	Monofocal and -centric on left and right side	Monofocal and -centric	not applicable
ER	0%	0%	0%	0%	0% on left and right side	1%	95%	100% on left and right side	100%	5%
PR	0%	5%	0%	0%	0% on left and right side	2%	90%	100% on left and right side	100%	5%
Her2	Positive (ICH 3+)	Positive (ISH positive)	Negative (IHC 1+)	Negative (IHC 0)	Negative (IHC 0) on left and right side	Negative (IHC 1+)	not applicable	not applicable	not applicable	Positive (ISH positive)
Ki-67	65%	80%	40%	60%	70% left, 85% on right	80%	not applicable	not applicable	not applicable	70%

N+/-= nodal positive or negative, Her2+/-= Her2 positive or negative, BCT= breast-conserving tumorectomy, SLN= sentinellymphnodectomy, MT= mastectomy, UL/BL= uni- versus bilaterality, MF/MC= multifocality or -centricity, ER= estrogen receptor, PR= progesterone receptor, Her2= Her2 status, Ki-67= Ki-67-proliferation-index

Standardized Prompt

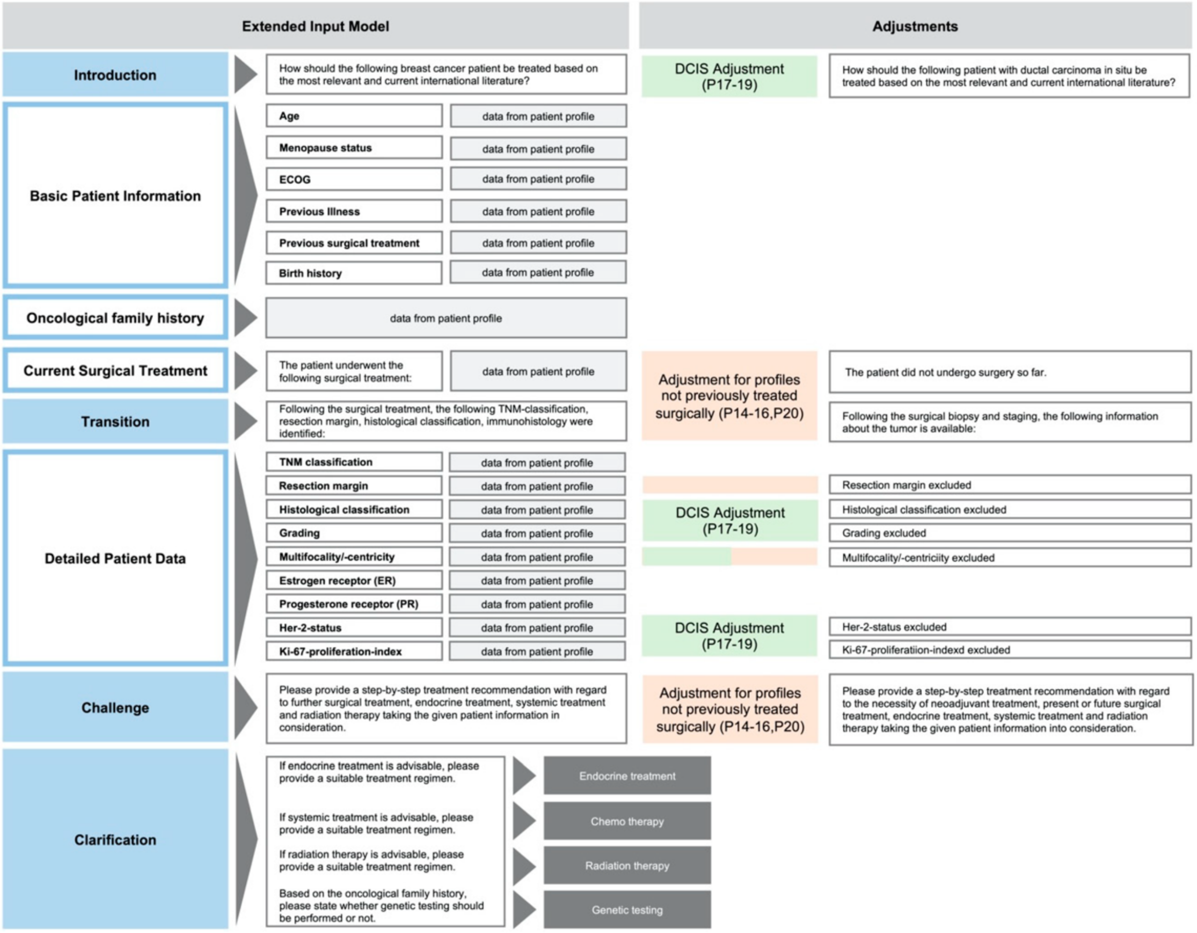

Supplement: Supplementary file 1 — Supplementary Material 1 [file 432_2024_5964_MOESM1_ESM.pdf]
