# Supplementary material for: Proof-of-concept study of a small language model chatbot for breast cancer decision support – a transparent, source-controlled, explainable and data-secure approach
Source: J Cancer Res Clin Oncol. 2024 Oct 9;150(10):451. doi: 10.1007/s00432-024-05964-3 (PMC11464535; doi:10.1007/s00432-024-05964-3)
Supplement: Supplementary file 2 — Supplementary Material 2 [file 432_2024_5964_MOESM2_ESM.pdf]

		BC-SLM					GPT-4					GPT-3.5				
PP		ST	ET	CT	RT	GT	ST	ET	CT	RT	GT	ST	ET	CT	RT	GT
Postmenopausal Luminal A N-	**1**	yes	yes	yes	yes	no	yes	yes	yes	yes	yes	yes	yes	yes	yes	no
Postmenopausal Luminal A N+	**2**	yes	yes	yes	yes	no	yes	yes	no	yes	yes	yes	yes	yes	yes	no
Premenopausal Luminal A N-	**3**	yes	yes	yes	no	yes	yes	yes	yes	yes	yes	yes	yes	yes	yes	yes
Premenopausal Luminal A N+	**4**	yes	yes	yes	yes	no	yes	yes	no	yes	yes	yes	yes	yes	yes	no
Postmenopausal Luminal B Her2- N-	**5**	yes	yes	yes	yes	yes	yes	yes	no	yes	yes	yes	yes	no	yes	yes
Postmenopausal Luminal B Her2- N+	**6**	yes	yes	yes	yes	yes	yes	yes	yes	yes	yes	yes	yes	yes	yes	no
Premenopausal Luminal B Her2- N-	**7**	yes	yes	yes	yes	no	yes	yes	yes	yes	no	yes	yes	no	yes	no
Premenopausal Luminal B Her2+ N+	**8**	yes	yes	yes	yes	yes	yes	yes	yes	yes	yes	yes	yes	yes	yes	yes
Postmenopausal Her2+ ER/PR- N-	**9**	yes	no	yes	yes	yes	yes	yes	yes	yes	yes	yes	no	yes	yes	no
Postmenopausal Her2+ ER/PR- N+	**10**	yes	yes	yes	yes	no	yes	yes	yes	yes	no	yes	yes	yes	yes	no
Premenopausal Her2+ ER/PR- N-	**11**	yes	yes	yes	yes	yes	yes	yes	yes	yes	yes	yes	yes	yes	yes	yes
Premenopausal Her2+ ER/PR- N+	**12**	yes	yes	yes	yes	yes	yes	yes	yes	yes	yes	yes	yes	yes	yes	yes
Postmenopausal Triple Negative N-	**13**	yes	yes	yes	yes	yes	yes	yes	yes	yes	yes	yes	yes	yes	yes	yes
Postmenopausal Triple Negative N+	**14**	yes	yes	yes	yes	yes	yes	yes	yes	yes	yes	yes	yes	yes	yes	yes
Premenopausal Triple Negative N-	**15**	yes	yes	yes	yes	yes	yes	yes	yes	yes	yes	yes	yes	yes	yes	yes

Premenopausal Triple Negative N+	**16**	yes	yes	yes	yes	yes	yes	yes	yes	yes	yes	yes	yes	yes	yes	yes
Postmenopausal DCIS, clear resection margin	**17**	yes	no	yes	no	yes	yes	no	yes	yes	yes	yes	no	yes	no	yes
Premenopausal DCIS, clear resection margin	**18**	yes	no	yes	yes	yes	yes	no	yes	yes	yes	yes	no	yes	yes	yes
Postmenopausal DCIS, narrow resection margin	**19**	yes	no	yes	no	no	no	no	yes	yes	no	no	no	yes	yes	no
Inflammatory Breast Cancer	**20**	yes	yes	yes	yes	no	yes	yes	yes	yes	yes	yes	yes	yes	yes	no
		**100.00%**	**80.00%**	**100.00%**	**85.00%**	**65.00%**	**95.00%**	**85.00%**	**85.00%**	**100.00%**	**85.00%**	**95.00%**	**80.00%**	**90.00%**	**95.00%**	**55.00%**

Yes = concordance/agreement with MTB, No = No concordance/agreement with MTB

BC-SLM = breast cancer small language model, ST = surgical re-excision, ET= endocrine treatment, CT=systemic or chemotherapy, RT= radiotherapy, GT= necessity for genetic counseling
